# A numerical investigation into the strength of offshore jacket platforms considering time-variant zonal corrosion subjected to alternative ship impacts

**DOI:** 10.1016/j.heliyon.2024.e40965

**Published:** 2024-12-06

**Authors:** Reza Zendehdel, Mohammad Reza Khedmati

**Affiliations:** Department of Maritime Engineering, Amirkabir University of Technology, No. 424, Hafez Avenue, Tehran, 15916-34311, Iran

**Keywords:** Jacket platform, Consecutive impacts, Time-variant zonal corrosion, Ultimate strength

## Abstract

In addition to the usual loads, fixed jacket offshore platforms can be exposed to accidental loads from ship collisions. Indentation of tubular components is a significant defect that occurs when a supply vessel collides with a jacket platform, which can affect the ultimate strength of the offshore platform. This paper performs a nonlinear dynamic analysis using ABAQUS software to evaluate the ultimate strength of a wellhead jacket platform and to investigate its structural response to two consecutive impacts from a 2700-ton ship. A total of 16 collision scenarios were simulated and analyzed. The research incorporates time-varying zonal corrosion for jacket platforms, as discussed by Yang et al., and employs beam and shell elements for the numerical modeling of the jacket platform, using a rigid model for the offshore supply vessel. The results indicate that the key factors affecting structural damage are the corrosion rate, consecutive impacts, and the shape of the impacting structure. Findings reveal that local indentation and local displacement are directly proportional to increases in the corrosion rate. However, the bending deformation parameter shows a different pattern: it has an inverse relationship with the corrosion rate and a direct relationship with the number of collisions. In sideway and stern collisions, the primary impact in each corrosion scenario has almost the same effect on the ultimate strength of the structure as the secondary impact in the previous corrosion scenario. However, in forecastle impacts, the effect of the secondary impact is greater than that of the primary impact in the subsequent corrosion scenario, equivalent to a 10-year increase in corrosion.

## Highlights

• The collision analysis adopts the principles of ductile design.
• To connect the beam and shell elements of the jacket, a kinematic coupling constraint was employed.
• Utilizes a time-varying zonal corrosion model for the wellhead platform.
• Nonlinear dynamic analysis to evaluate the ultimate strength of the platform under the impact of two consecutive ship collisions.
• Key factors affecting structural damage include the corrosion rate, consecutive impacts, and the shape of the impacting structure.

## 1 Introduction

### 1.1 Statement of the problem

**Fig. 1:**
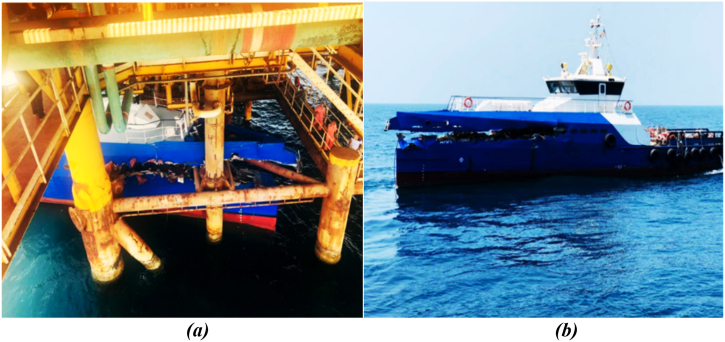
(a) Collision of a high-speed vessel with South Pars platform 12B; **(b)** The extent of damage to the ship [1].

The continuous growth of the offshore oil and gas industry and society's reliance on hydrocarbons have led to significant advancements in the construction and design of marine structures and offshore platforms. Fixed offshore jacket platforms primarily consist of steel tubular members, which are essential elements subjected to various loads including bending, tensile, compressive, and torsional forces. Indentation is recognized as one of the most critical defects that can occur in these tubular members, affecting the structural integrity of the platform. The structural integrity of offshore jacket platforms can be compromised by several factors such as corrosion from exposure to the corrosive marine environment, connection cracking, fatigue from wave impacts, and denting from ship collisions. The circular cross-section of tubular members is particularly vulnerable to oval deformation and local indentation caused by lateral impacts, resulting in a significant reduction in axial and bending capacities, which ultimately weakens the platform's ultimate strength. Ship collisions with fixed jacket platforms pose a serious risk to structural integrity and can potentially result in catastrophic failures. Fig. 1(a) illustrates a high-speed vessel colliding with the South Pars platform 12B, located in the South Pars gas field. According to Fig. 1(b) this collision caused severe damage, primarily to the ship's structure, leading to hull rupture.

**Fig. 2:**
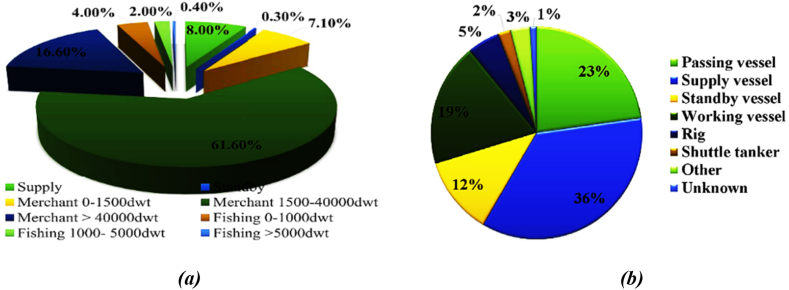
(a) Overall distribution of ship traffic within the 10 nm exclusion zone; **(b)** accident statistics based on vessel type [2].

With the continuous growth of marine transportation and the subsequent increase in marine traffic, the risk of ships colliding with offshore platforms has escalated. Numerous studies have been conducted to examine the types of ships involved in these collisions and the frequency of such incidents. In 2010, the Marine Accident Database compiled and published data on the distribution of ship types involved in ship/platform accidents. According to the collected data, offshore supply vessels were identified as the ship type with the highest percentage of collisions with jacket platforms. Furthermore, it was observed that the majority of ships passing within a 20 km radius of marine platforms are support vessels and commercial ships weighing at least 40,000 tons. Fig. 2(a-b) provides a visual representation of the main types of ships that collide with or pass near offshore platforms.

### 1.2 Review of previous works

The collision of ships with fixed offshore platforms is a critically important topic that has been extensively studied for several decades. Since 1980, numerous researchers have focused on understanding the dynamics of such incidents. Over the years, both researchers and industry professionals have conducted various experiments and studies to comprehend the mechanics of impact and assess the structural integrity of platforms during ship-platform collisions. Established standards such as HSE [3], DNV GL [4], ABS [5], and NORSOK [6] have also provided design criteria specifically addressing collision issues. Numerous studies have concentrated on the problem of ship collisions with fixed offshore platforms. Jin et al. [7] introduced a non-linear dynamic analysis method to determine impact actions by analyzing forensic evidence from damaged components. They assessed the overall damage effects on platform structures through simulations of impacts using a triangle impulse load at varying collision contact times. Naderi and Aghakouchak [8] investigated the potential increase in platform vulnerability due to damage to members in the splash zone following a ship collision. They evaluated the damaged structure's capacity to dissipate impact energy and compared it to its intact condition, providing insights into platform safety and structural integrity.

Khedmati and Nazari [9] conducted a numerical investigation to analyze the structural behavior of preloaded tubular members subjected to lateral impact loads using the finite element method. They also examined the effects of preloading on the buckling and ultimate strength of laterally impacted tubes. Their findings revealed that increasing preloading reduces the ultimate strength, causing the members to collapse under lower loads. In a related study, Li et al. [10] focused on ship-jacket platform collisions and studied the elastic and plastic responses of the jacket platform. They developed simulation models for both ductile and rigid supply vessels, as well as for two typical jacket platforms. Their results indicated that the NORSOK rule underestimates the resistance to certain indentations due to an inaccurate description of the column deformation mode.

Sumiwi et al. [11] investigated the impact of graded collision velocities of a 2500-ton supply vessel on the local and global structural damage of the CONOCO BELANAK wellhead platform during a collision event. Their findings showed that through push-over analysis, the jacket structure would collapse after a series of fifty incremental collisions. Travanca and Hao explored the prediction of dynamic responses of offshore steel platforms to high-energy impacts from standard supply vessels. They introduced a methodology to establish simplified equivalent systems for efficient structural response analysis and validated their approach by comparing the results with explicit nonlinear finite element simulations [12]. In a subsequent study in 2015, Travanca and Hao shifted their focus to energy dissipation during high-energy ship-offshore jacket platform collisions. The researchers evaluated various models under different collision scenarios, analyzing strain energy dissipation concerning varying ship/installation strength ratios [13].

In 2014, Buldgen et al. conducted a study focusing on estimating the crushing resistance of an oblique cylinder impacted by the stem of a striking ship. Their research aimed to assess the crashworthiness of offshore wind turbine jackets through the development of a simplified analytical method [14]. Additionally, Dahiwalker and Solanki investigated the behavior of jacket structures following collision events. Their study involved comparing results obtained from ANSYS with a theoretical formulation based on a rigid plastic mechanism approach. The findings indicated that columns with substantial local indentations experienced a significant reduction in plastic collapse capacity compared to the ideal load [15]. Rigueiro et al. [16] conducted a comprehensive numerical analysis on the response of the Merluza-1 offshore steel platform in Brazil to impact loading from 5000-ton stiff and soft ships. Their study focused on quantifying the deformation and energy dissipation in both the ship and the platform during localized collision events. In a related study, Yu and Amdahl [17] investigated the structural response of offshore tubular members subjected to impacts from vessels at the bow and stern using the nonlinear finite element code LS-DYNA. Their research included a parametric study on denting mechanics, considering factors such as tubular member length, diameter, and wall thickness. This study provided valuable insights into the behavior of tubular members under varying impact conditions.

Mujeeb-Ahmed et al. [18] pioneered practical modeling techniques for assessing structural crashworthiness in collisions between fixed-type offshore platforms and offshore supply vessels. Their computational models utilized a nonlinear finite element method to capture large deformations and strains in both the ship and platform, accounting for dynamic material effects such as strain rate, dynamic fracture strain, and the influence of surrounding water. Qasim and Hasan investigated the impact of soil-pile interaction on the response of platforms to lateral impact loads through finite element simulations conducted using ABAQUS software [19]. Their research focused on analyzing pile lateral displacement, pile twist angle, pile shear force distribution, pile bending moment distribution, and deck slab displacement, providing insights into the complex behavior of platforms under lateral loading conditions. Mujeeb-Ahmed and Paik introduced a novel Quantitative Risk Assessment model for evaluating collisions between offshore supply vessels and jacket-type offshore platforms. They conducted detailed vessel motion analyses to determine collision load characteristics by selecting fifty collision scenarios using probabilistic sampling techniques [20]. In a related study, Hao et al. [21] used the finite element software ABAQUS to simulate ship collision damage processes on jacket foundations under various combinations of ship mass, initial velocity, and collision angle. Their findings revealed that the bearing capacity of the damaged structure decreases with increasing ship mass or impact speed, with no significant correlation observed with the impact angle.

### 1.3 Necessity of the present work and its outlines

Numerous research efforts have explored the collision dynamics between ships and fixed offshore platforms across various disciplines. Previous studies in the field of ship/jacket collisions have primarily focused on evaluating the platform's strength concerning corrosion, particularly in members located in the splash zone. However, limited attention has been given to assessing corrosion in other areas of jacket platforms, including tidal, submerged, and atmospheric zones. Additionally, existing studies have predominantly examined the effects of the initial collision, often overlooking subsequent collisions, such as second or even third impacts. The interaction between waves, wind forces, and vessels can lead to vessel movement and secondary collisions with offshore platforms. Hence, it is crucial to consider repeated impacts in the design of offshore structures. This study aims to address these gaps in the literature by exploring the impact of multiple collisions on the structural integrity of fixed jacket platforms. The objectives of this study are outlined as follows.

• Conduct a nonlinear dynamic analysis using a ductile design approach to evaluate the ultimate strength of the S1 wellhead platform in the Iran Salman field and investigate the structural response to two consecutive ship impacts.
• Incorporating time-varying zonal corrosion for jacket platforms, as discussed in the research by Yang et al. [22].
• Utilizing beam and shell elements for numerical modeling of a jacket platform and employ a rigid model for an offshore supply vessel in the ABAQUS software.

## 2 Corrosion of jacket platform structures

### 2.1 Corrosion models for jacket platform structures

The degradation of tubular components in offshore structures due to corrosion is highly variable, owing to the complex and uncertain nature of this phenomenon. Corrosion models developed over the years have shown that changes in the structural members of marine platforms do not always follow a linear pattern. These models can be categorized into experimental, phenomenological (qualitative analysis based on experimental data), probabilistic, and physical models. Empirical models rely on historical data or measurements of corrosion loss, while physical models are based on the actual corrosion process [23,24]. Generally, corrosion of fixed jacket platform members can be classified into two categories: general corrosion and local corrosion. Researchers have also categorized corrosion prediction models for marine steel structures into linear and non-linear types, as outlined in Table 1. The table reviews all corrosion models for marine structures based on their introduction year and provides a brief description of each model. Notably, the research under consideration focuses on the predictive model of Yang et al. for fixed jacket platforms [22].

**Table 1:** Corrosion models for marine structures.

Corrosion Model (Year)	Type of Model		Equation	Description of Model
Southwell et al., 1965 [25]	Linear		d(T)=0.076+0.038T	Their study is grounded in realistic data sources and their models tend to overestimate corrosion loss in the early stages of exposure.
	Bilinear		$d\left(T\right)=\left\{ \begin{array}{c} 0.09T,0\leq T<1.46\;years\\ 0.76+0.038T,1.46\leq T<16\;years \end{array}\right.$	
Guedes Soares; 1988 [26]	Linear		$t_{k}=t-{kt}_{*}$	The reduction in thickness of the structural member demonstrates a linear correlation with time.
Yamamoto and Ikegami; 1998 [27]	Nonlinear		$d\left(T\right)=C_{1}{\left(T-T_{0}-T_{t}\right)}^{C_{2}}$	Their study relies on a probabilistic model which exhibits a high level of complexity and does not adequately align with the available data.
	Pitting Model (Growth Pattern of the Pitting Points)		$z\left(t\right)=a\tau^{b}$	
Melchers Based on the Southwell Study; 1999 [28]	Nonlinear		$d\left(T\right)=0.84T^{0.823}$	This model offers mathematical sophistication but struggles to align with existing data.
	Trilinear		$d\left(T\right)=\left\{ \begin{array}{c} 0.170T,0\leq T<1\;years\\ 0.152+0.0186T,1\leq T<8\;years\\ -0.364+0.083T,8\leq T<16\;years \end{array}\right.$	
	Power Law		$d\left(T\right)=0.1207T^{0.6257}$	
Guedes Soares and Garbatov; 1999 [29]	Nonlinear		$d\left(T\right)=d_{\infty }\left[1-e^{\left(-\frac{T-T_{c}}{T_{t}}\right)}\right]$	The benefit of this model lies in its practical application as a corrosion wastage tool. However, it lacks a clear distinction between the final two phases.
Lutes et al., 2001 [30]	Uniform Loss of Wall Thickness-Symmetric Cross-Section		$t=t_{0}-{Cr}_{\max}$	In this model, the impact of asymmetry is examined by analyzing a severe scenario where external corrosion is highly asymmetric and varies along the member's length to maximize the bending moment caused by eccentricity.
	Nonuniform Loss of Wall Thickness-Asymmetric Cross-Section		$t\left(\theta_{1}\right)=t_{0}-\frac{{Cr}_{\max}\left|\theta_{1}\right|}{\pi },-\pi \leq \theta_{1}\leq \pi$	
Paik et al., 2003 [31]	Nonlinear		$d\left(T\right)=C_{1}{\left(T-T_{cl}\right)}^{C_{2}}$	This model serves as a practical tool for assessing corrosion wastage, but it has drawbacks, including a lack of clear guidance on selecting the appropriate curve and unrealistic assumptions about the absence of corrosion damage during the transition phase.
Qin and Cui; 2003 [32]	Nonlinear		**_**	The flexibility and explanatory power of corrosion models through parametric adjustments are notable advantages. However, these models have drawbacks, such as the lack of definitive conclusions regarding accuracy and the reliance on assumed data for model fitting.
Melchers and Jeffrey; 2008 [33]	Nonlinear		**_**	This model is complex and requires an explanation of the actual corrosion mechanism.
Yang et al., 2019 [22]	Time-Variant Zonal	Atmospheric	d(T)=0.05T	An accurate corrosion model has been developed for the platform splash zone using the Weibull distribution based on real data. For the other four zones, an approximate linear corrosion model has been provided due to the lack of available measured data.
		Splash	$d\left(T\right)=10\left\{1-\exp\left[-{\left(\frac{T}{20.9153}\right)}^{1.8052}\right]\right\}$	
		Tidal	d(T)=0.1244T	
		Submerged	d(T)=0.0516T	
		Subsoil	d(T)=0.05T	

### 2.2 Corrosion zones of jacket platform structures

**Fig. 3:**
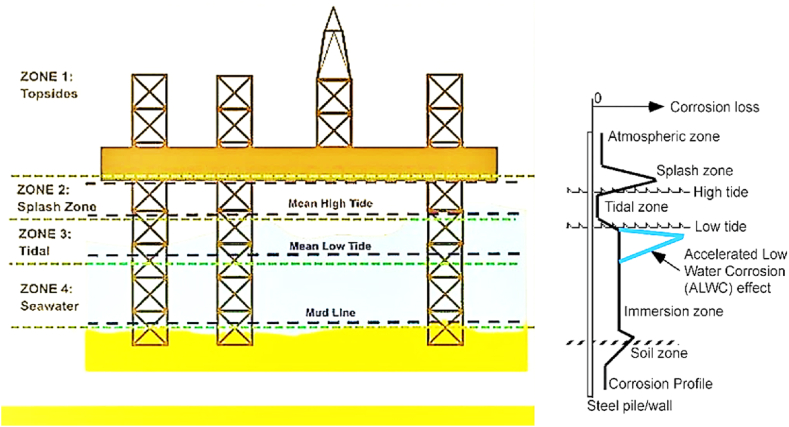
Illustration showing the zones of fixed offshore platforms at sea and the corrosion rates across the five areas [34].

In general, the fixed offshore platform structure can be categorized into five different corrosion zones based on their location in oceans and seas. These zones, as depicted in Fig. 3, range from the platform deck to the seabed.

• Atmospheric zone
• Splash zone
• Tidal zone
• Submerged zone
• Subsoil zone

The corrosion behavior of metals varies depending on the environmental conditions to which they are exposed. In fixed offshore platform structures, most metals and alloys are susceptible to corrosion when in contact with seawater and oxygen.

### 2.3 Time-variant corrosion model

The permissible corrosion rate of steel tubular members in offshore structures varies across different service periods. Yang et al. [22] developed a corrosion model for offshore platforms known as the time-variant zonal corrosion model, which accounts for location and time-dependent variations. Based on measured corrosion data from the No. 8 Bohai Jacket Platform, an ex-service platform, it is evident that the corrosive depth of the steel tubular members in the splash zone typically progresses slowly at the initial stage, accelerates in the intermediate stage, and decelerates in the final stage [35].

**Fig. 4:**
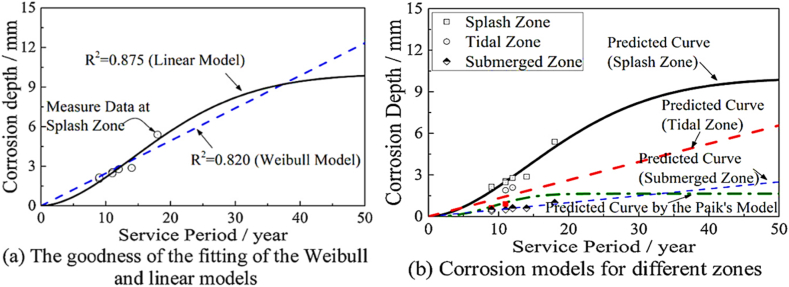
The zonal corrosion models of offshore platforms based on the Yang et al. study [22]: **(a)** Fitting accuracy of the Weibull model and the linear model; **(b)** Corrosive depth of tubular members.

Yang et al. [22] characterized the corrosion development in the splash zone of the Bohai Jacket Platform using measured data and experimental results from Paik et al. [36], employing a Weibull distribution. In the absence of measured data, a linear distribution was used to simulate corrosion development in the remaining four zones. The fitting accuracy of the Weibull model and the linear model proposed by Yang et al. are compared and illustrated in Fig. 4(a). Using the fitted outcomes from Yang et al., the corrosive depth of tubular members in jacket platforms can be determined using the equations provided in Table 1 and Fig. 4(b).

### 2.4 Corrosive depths of tubular members

The depth of corrosion in the tubular members of a jacket platform structure can be determined across five distinct zones, as outlined in Table 2. According to the equation detailed in Table 1, the external diameter of a corroded tubular member is calculated by subtracting twice the depth of corrosion from the initial external diameter. Comparing the equation derived by Yang et al. reveals that the depth of corrosion in the tubular members within the splash and tidal zones exceeds that predicted by the corrosion model of Paik et al. Furthermore, Table 2 shows that the corrosion levels of members in the atmospheric, submerged, and subsoil zones of the jacket platform are significantly lower than those in the splash and tidal zones. For the service period ranging from 8 to 32 years, the corroded depth of members in these three zones, according to Yang et al.'s model, is less than that projected by Paik et al.'s model.

**Table 2:** Change in thickness of structural members of the jacket at different zones [22].

Time-in Service (*Year*)	Change in Thickness of Structural Members of the Jacket (*mm*)					
	Based on the Zonal Corrosion Model of Yang et al. [22]					Based on the Corrosion Model of Paik et al.
	Atmospheric Zone	Splash Zone	Tidal Zone	Submerged Zone	Subsoil Zone	
0	0	0	0	0	0	0
2	0.1	0.143	0.249	0.103	0.1	0.02
4	0.2	0.492	0.498	0.206	0.2	0.11
6	0.3	0.996	0.746	0.31	0.3	0.3
8	0.4	1.617	0.995	0.413	0.4	0.56
10	0.5	2.32	1.244	0.516	0.5	0.86
12	0.6	3.071	1.493	0.619	0.6	1.14
14	0.7	3.84	1.742	0.722	0.7	1.36
16	0.8	4.602	1.99	0.826	0.8	1.5
18	0.9	5.336	2.239	0.929	0.9	1.58
20	1	6.024	2.488	1.032	1	1.62
22	1.1	6.657	2.737	1.135	1.1	1.63
24	1.2	7.225	2.986	1.238	1.2	1.64
26	1.3	7.726	3.234	1.342	1.3	1.64
28	1.4	8.161	3.483	1.445	1.4	1.64
30	1.5	8.531	3.732	1.548	1.5	1.64
32	1.6	8.841	3.981	1.651	1.6	1.64
34	1.7	9.096	4.23	1.754	1.7	1.64
36	1.8	9.304	4.478	1.858	1.8	1.64
38	1.9	9.471	4.727	1.961	1.9	1.64
40	2	9.602	4.976	2.064	2	1.64
42	2.1	9.704	5.225	2.167	2.1	1.64
44	2.2	9.783	5.474	2.27	2.2	1.64
46	2.3	9.842	5.722	2.374	2.3	1.64
48	2.4	9.887	5.971	2.477	2.4	1.64
50	2.5	9.92	6.22	2.58	2.5	1.64

According to the zonal corrosion model, the total mass loss in jacket platforms due to corrosion is similar to that predicted by the Paik model if the service period is less than approximately 18 years. However, for service periods exceeding 18 years, a significant variation in the predicted amount of steel loss is observed between the two corrosion models [22]. Moreover, the time-varying zonal corrosion model studied by Yang et al. shows a faster increase in predicted steel loss compared to the commonly used uniform corrosion model, even when using the same corrosion parameters.

## 3 Methodology

### 3.1 Design principles and method of analysis

**Fig. 5:**
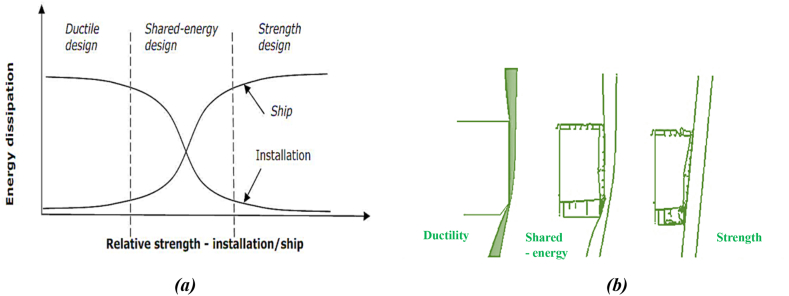
(a) Energy dissipation for strength, ductile and shared-energy design; **(b)** Schematic diagram of structural deformation [37].

The analysis and design of fixed jacket platform structures against ship collisions are divided into three regimes in normal mode, based on the relative strength of the platform and ship structures. These three design principles, as illustrated in Fig. 5(a), describe how energy is dissipated between the impacting and impacted structures [37].

• **Strength design**: The ship's structure has limited strength, while the jacket platform has sufficient strength to withstand minor deformations without experiencing significant plastic deformations that could potentially damage the ship (Fig. 5(b)). The ship structure is designed to change its shape and absorb a significant portion of the impact energy. This approach is commonly used in the design of ship hulls to withstand collisions.
• **Ductility design:** The body of the impacting ship is assumed to be infinitely rigid (Fig. 5(b)), while the platform undergoes significant plastic deformations during impact, leading to considerable energy dissipation. The shape of the indentation is largely influenced by the rigid geometry of the ship structure, and energy absorption can be analyzed using plastic methods.
• **Shared-energy design**: Both the platform and the ship structure contribute significantly to energy dissipation. The magnitude and distribution of impact force rely on the deformation of both the ship and platform structures.

In this study, the non-linear finite element analysis (NLFEA) method was used to assess the strength of offshore jacket platforms, considering time-variant zonal corrosion under alternating ship impacts. The analysis follows the ductility design principle, assuming that the ship does not deform. A dynamic/explicit solver within SIMULIA's ABAQUS/CAE software was employed to perform the collision simulation. This research does not account for additional external environmental factors such as wind and waves, as the primary focus is on the alternating forces exerted by the ship.

Three different elements were used in modeling the fixed jacket platform and ship structure. Shell elements were applied to the fixed jacket platform members in the collision and near-collision zones, while beam elements were used for the remaining platform members to reduce problem-solving time in the software. The ship structure was assumed to be rigid. The widely-used shell element S4R in Abaqus software was employed to model platform members in impact areas and their vicinity. This four-node element, part of the plate group, is well-known for its suitability and common usage in both general and complex plate analysis applications. It features a reduced integration formulation and effectively controls shear locking (hourglass phenomenon). For the other fixed platform members, the B31 beam element was utilized to streamline problem-solving time in the modeling software. The B31 element serves as a beam component for three-dimensional structures. To connect the beam and shell elements of the platform structure, a coupling constraint with a kinematic type was employed in Abaqus, allowing the coupling of all degrees of freedom. When deformation due to external loads is not desired, a rigid component can be applied to the structure's body. In the modeling, the ship structure's body was considered rigid, using the R3D4 element type.

### 3.2 Physical models and their characteristics for the jacket platform and the supply vessel

**Fig. 6:**
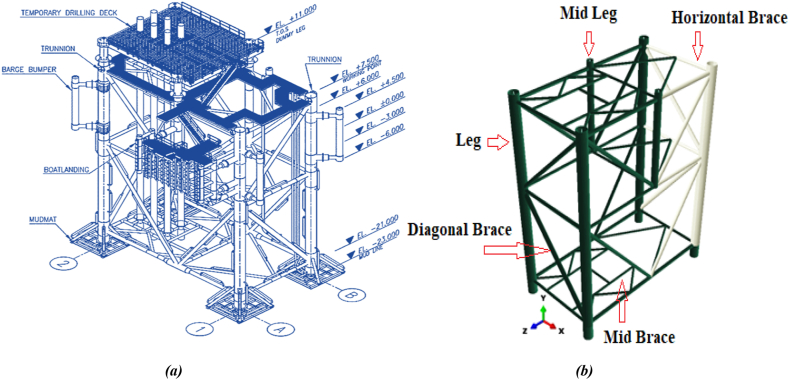
(a) Schematic image of fixed jacket platform S1 of Salman oil field; **(b)** simulated jacket platform in Abaqus software.

The jacket platform investigated in this study is the S1 wellhead platform located in the Salman common field within the Persian Gulf waters. The Salman oil and gas field is an active oil production complex situated 144 km south of Lavan Island in the Persian Gulf. The wellhead platform under study features four legs, four piles, and three riser pipes. However, in the simulation of the jacket platform, the riser pipes were not modeled. One simplifying assumption made in this research was to use equivalent thickness and diameter for the platform legs instead of modeling its piles. The dimensional specifications, including the thickness and diameter of the fixed jacket platform structure members used in the modeling, are detailed in Table 3. Fig. 6(a) schematically depicts the Salman S1 fixed oil platform, while Fig. 6(b) illustrates the simulated platform.

**Table 3:** Diameter and wall thickness of jacket platform of members.

Structural Members		Diameter (*mm*)	Wall Thickness (*mm*)
Elevation of Row 1& 2	Leg	1334.6	45
	Horizontal Brace	406.4	19.05
	Diagonal Brace	508	25.4
	Horizontal Brace	406.4	19.05
	Diagonal Brace	610	25.4
	Horizontal Brace	406.4	19.05
Elevation of Row A & B	Leg	1334.6	45
	Mid Leg	914.4	25.4
	Horizontal Brace	508	25.4
	Diagonal Brace	508	19.05
	Diagonal Brace	508	19.05
	Horizontal Brace	406	19.05
	Diagonal Brace	457	25.4
	Diagonal Brace	457	25.4
	Horizontal Brace	508	25.4
Plan at Elevation +6.0 & −21.0	All Mid Braces	323.5	12.7
Plan at Elevation −6.0	All Mid Braces	355.6	12.7

**Fig. 7:**
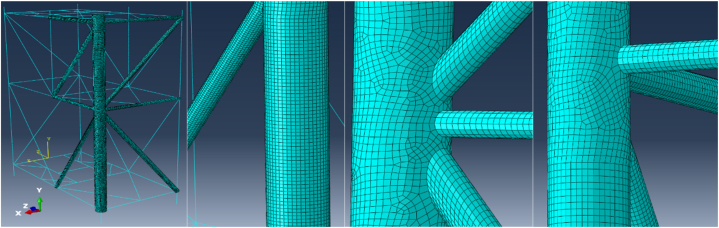
Finite element mesh for jacket platform.

The structures were segmented to facilitate a structured mesh with elements of uniform size. The element size was determined based on data from previous studies that used the same structures for simulation, as documented by Le Sourne et al., 2015 [38]. It was observed that reducing the element size below 0.15 m did not significantly affect the results. A coarser mesh was applied to the rigid components to reduce both CPU time and data size. Fig. 7 shows the finite element mesh for the offshore jacket platform.

**Fig. 8:**
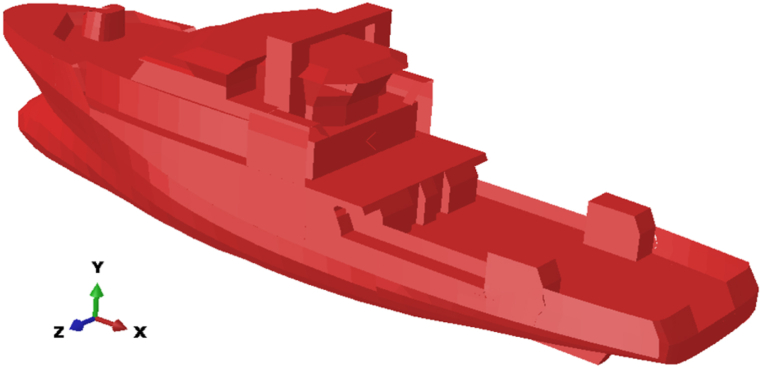
Simulated multipurpose supply vessel model in Abaqus software.

Based on statistical data on collisions between ships and platforms, the ship model considered in this study is a multi-purpose offshore supply vessel. These vessels are more common around fixed offshore platforms compared to other types of vessels. Fig. 8 shows an image of the multi-purpose supply vessel model used in this research, and Table 4 provides its full specifications.

**Table 4:** Main particulars of the multi-purpose offshore support vessel with steel hull and superstructure.

Particular	Value
Length (*m*)	62
Beam (*m*)	12
Draft (*m*)	4.5
Displacement (*tons*)	2700
Max Speed (k*nots*)	16
Range (S*ea Mile*)	3500

### 3.3 Mechanical properties and behavior of the materials

**Fig. 9:**
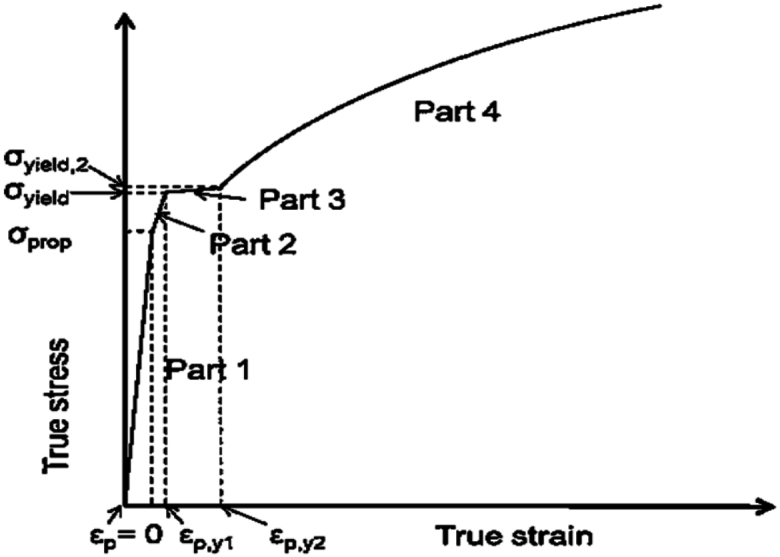
Stress-strain curve based on DNVGL-RP-C208 (2016).

In collision analyses involving steel structures, it is crucial to consider both strain hardening and strain-rate hardening. This section outlines the material model adopted, in accordance with the guidelines from DNVGL-RP-C208 [39]. Fig. 9 shows the true stress-strain curve for plastic strain, along with the corresponding parameters.

Up to $\varepsilon_{p,y2}$ the stress-strain relationship follows a piecewise-linear relationship. The yield plate is located within $\varepsilon_{p,y1}<\varepsilon <\varepsilon_{p,y2}$. After $\varepsilon_{p,y2}$ the stress-strain relationship follows a hardening model which is given in Eq. (1) (The parameters *K* and *n* in the stress-strain relationship are obtained from Eq. (2) and Eq. (3), respectively.):

(1) $$\sigma =K.\left(\varepsilon_{p}+{\left(\frac{\sigma_{yield2}}{K}\right)}^{\frac{1}{n}}-\varepsilon_{p,y2}\right)$$

(2) $$K=\sigma_{UTS}.{\left(\frac{e}{n}\right)}^{n}$$

(3) $$n=\ln\;1+\varepsilon_{UTS}$$

The parameters in Eq. (1) depend on the material type, thickness, and resistance. According to the specifications of the offshore wellhead platform project, the jacket structure uses low-carbon steel S355. Specific material parameters are detailed in Table 5. As per NORSOK N-004 [40], the critical strain is influenced by factors such as strain rate, the presence of strain concentrations, defects, and material toughness. NORSOK has proposed a critical strain value of 0.15 for steel type S355.

**Table 5:** Low resistance values for S355 based on DNVGL-RP-C208 (2016) [39].

S355				
Thickness (*mm*)	t≤16	16<t≤40	40<t≤63	63<t≤100
E (*MPa*)	210000	210000	210000	210000
$\sigma_{\text{prop}}$ (*MPa*)	320.0	311.0	301.9	284
$\sigma_{\text{yield}}$ (*MPa*)	357.0	346.9	336.9	316.7
$\sigma_{yield2}$ (*MPa*)	366.1	355.9	345.7	323.8
$\varepsilon_{p\_y1}$	0.004	0.004	0.004	0.004
$\varepsilon_{p\_y2}$	0.015	0.015	0.015	0.015
K (*MPa*)	740	740	725	725
n	0.166	0.166	0.166	0.166

### 3.4 Contact, boundary, and loading conditions

**Fig. 10:**
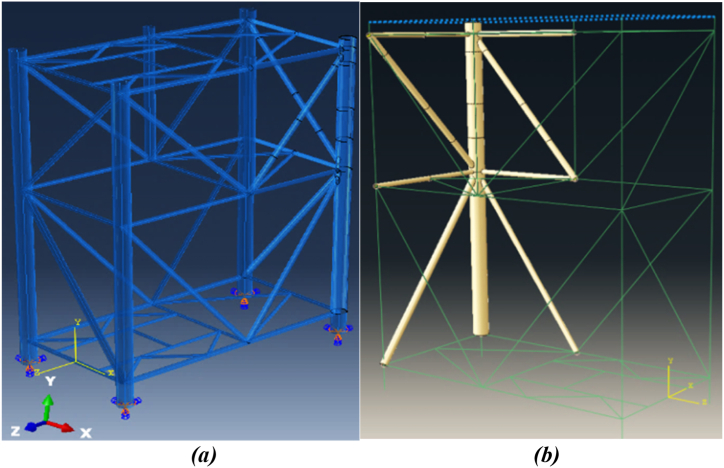
(a) Fixed boundary conditions for the jacket platform; **(b)** creating rigidity for the foundations using wire features in the interaction module.

Generally, one of three approaches—soil modeling, spring-dashpot modeling, or end-girder modeling—can be used to define the end boundary conditions of fixed marine platforms. In this research, the boundary conditions are simplified by assuming that the platform legs are fixed at their ends (Fig. 10(a)). The loading consists of forces from drilling operations as well as dead and live loads from the platform's upper decks. Rather than modeling the upper decks, these loads are directly applied and concentrated on the legs. The total force transferred to the foundation from the upper decks is 300 tons. To account for the rigidity that the upper decks provide to the foundation, wire features were used in the interaction module, enhancing the realism of the boundary conditions and improving the reliability of the results (Fig. 10(b)).

**Fig. 11:**
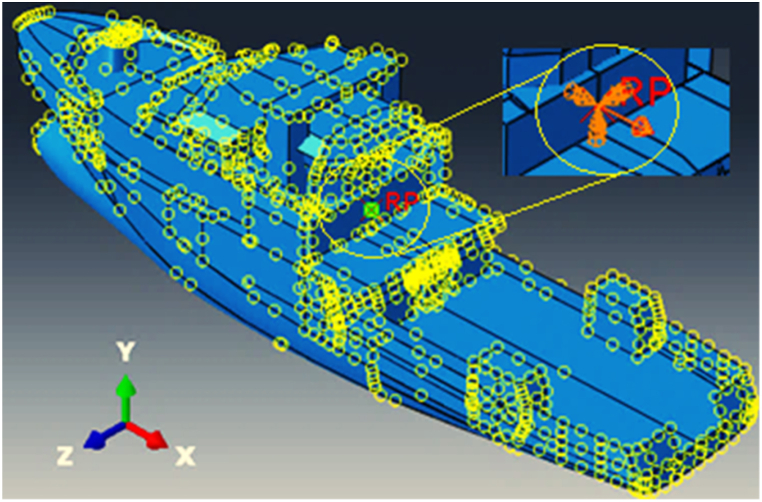
Application of the initial condition of the vessel through the reference point.

A reference point is used to apply the ship's weight and initial conditions, such as the initial speed of the collision, ensuring that all nodes comply with the specifications of the reference point. The boundary conditions at this reference point, which control the movement of the entire ship structure, are only open in the direction of movement, while other degrees of freedom are constrained (Fig. 11). Hard-contact algorithms are employed to model the interaction between the ship and the platform, with an amplitude defined for repetitive impacts of the ship. Additionally, a dynamic friction coefficient of 0.3 is applied during contact.

### 3.5 Corrosion model

Two types of corrosion, general and local, are used to evaluate the strength and behavior of fixed jacket platform structures. Typically, uniform corrosion is used in modeling, where the thickness reduction of all members across different zones is considered uniformly. The allowable corrosion limit of platform steel members in various zones and service periods changes over time. This research employs a time-variant zonal corrosion model based on the relationships presented by Yang et al., in 2019 [22]. The corrosion model aligns with the service life of the fixed jacket platform and serves as a criterion for collision scenarios. Corrosion is considered for four different service periods: zero years (representing a period without corrosion defects), 10 years, 20 years, and 30 years.

### 3.6 Collision scenarios and location of impacts

The collision scenarios considered in this research are based on recent incidents involving ship-platform collisions. A total of 16 scenarios have been analyzed, taking into account factors such as the corrosion model, collision area, speed, and direction. These scenarios include collisions involving the forecastle, side, and stern with the jacket leg, as well as the forecastle colliding with the brace member. Collision velocities are determined according to the accidental limit state specified by NORSOK N-003 (2017) regulations, with a velocity of 3 m/s for forecastle collisions and 2 m/s for side and stern collisions [41]. Generally, most collisions occur at altitude levels ranging from −5.5 to +8.2 m relative to the lowest astronomical tide level of the fixed jacket platform. The altitude level of a ship's collision with fixed jacket platforms under the environmental conditions of the Persian Gulf is calculated according to the minimum and maximum levels using Eq. (4) and Eq. (5):

(4) $$\mathrm{Min}\;Level=LAT+HAT+\frac{2}{3H_{\mathrm{Max}}}+Storm\;Surge+Subsidence+\mathrm{Min}\;Draft=LAT+1.9+4.5+0.2+0.3+1.5=LAT+8.4$$

(5) $$\mathrm{Max}\;Level=LAT-\frac{1}{3H_{\mathrm{Max}}}-\mathrm{Max}\;Draft=LAT-2.2-1.5=LAT-3.7$$

**Fig. 12:**
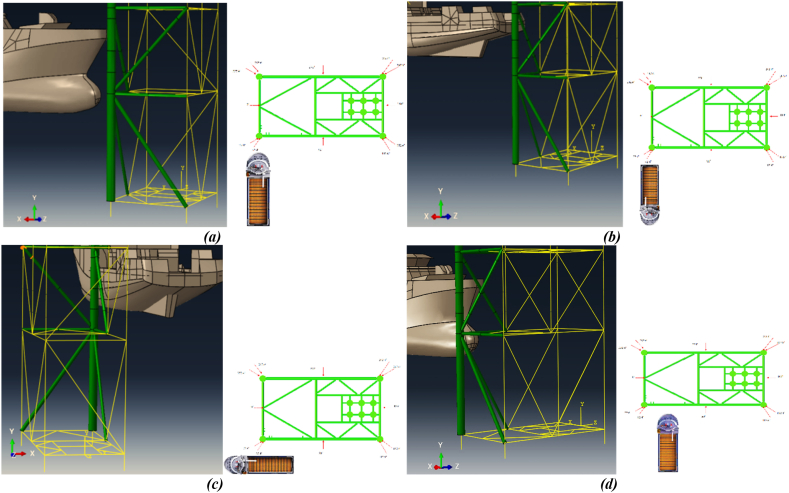
(a) Collision of the forecastle with the leg; **(b)** collision of the stern with the leg; **(c)** collision of the sideway with the leg; **(d)** Collision of the forecastle with the brace.

Table 6 outlines all the possible collision scenarios between the ship and the fixed platform. Additionally, these scenarios are illustrated in Fig. 12(a-d).

**Table 6:** Collision scenarios.

Collision Scenario	Collision Direction	Initial Velocity (*m/s*)	Collision Angle (*Degree*)	Collision Zone	Time in Service (*Year*)	Collision Altitude Level
**1**	Head on bow	3	90	Leg	0-Uncorroded	Splash (+2.5 *m*)
**2**	Head on bow	3	90	Leg	10	Splash (+2.5 *m*)
**3**	Head on bow	3	90	Leg	20	Splash (+2.5 *m*)
**4**	Head on bow	3	90	Leg	30	Splash (+2.5 *m*)
**5**	Sideway	2	0	Leg	0-Uncorroded	Splash (+2.5 *m*)
**6**	Sideway	2	0	Leg	10	Splash (+2.5 *m*)
**7**	Sideway	2	0	Leg	20	Splash (+2.5 *m*)
**8**	Sideway	2	0	Leg	30	Splash (+2.5 *m*)
**9**	Stern	2	90	Leg	0-Uncorroded	Splash (+2.5 *m*)
**10**	Stern	2	90	Leg	10	Splash (+2.5 *m*)
**11**	Stern	2	90	Leg	20	Splash (+2.5 *m*)
**12**	Stern	2	90	Leg	30	Splash (+2.5 *m*)
**13**	Head on bow	3	90	Brace	0-Uncorroded	Splash (+1.5 *m*)
**14**	Head on bow	3	90	Brace	10	Splash (+1.5 *m*)
**15**	Head on bow	3	90	Brace	20	Splash (+1.5 *m*)
**16**	Head on bow	3	90	Brace	30	Splash (+1.5 *m*)

### 3.7 Validation of the numerical procedure

To validate the research methodology and demonstrate the accuracy of the ABAQUS program results, preliminary validation was performed. In this study, the entire jacket platform structure was not modeled entirely with shell elements; instead, shell elements were applied only in the collision areas, while the remaining components were modeled using beam elements with degrees of freedom coupled at the connection points. This approach was validated by re-modeling the ship collision with an offshore wind turbine structure as studied by Moulas et al., in 2017 [42]. The simulation approach used in the present study was then compared with the results from Moulas et al.'s research.

**Fig. 13:**
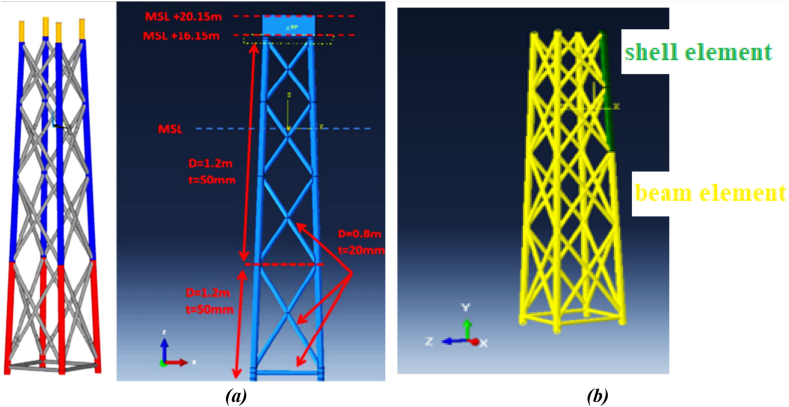
(a) Simulated offshore wind turbine foundation in Moulas et al.’s study [42]; **(b)** simulated offshore wind turbine foundation in the current study.

The offshore wind turbine foundation in Moulas et al.'s study is based on the project by Fabian Vorpahl et al., in 2011 [43]. The wind turbine foundation structure features four legs, each with a diameter of 1.2 m and a thickness of 50 mm. All bracing members have a diameter of 0.8 m and a thickness of 20 mm. The concrete block positioned on top of the jacket, which transmits the applied loads, is considered to weigh approximately 666 tons in their study. The members of the jacket structure in their study were all modeled using SR4 shell elements. The material specifications for the members are based on S355 steel, with Johnson-Cook's law used to define the material properties. The Johnson-Cook coefficients for S355 steel were sourced from the study by Gamarino et al. [44]. Fig. 13(a) shows the jacket structure from Moulas et al.'s study, while Fig. 13(b) shows the jacket structure in the current simulation.

**Fig. 14:**
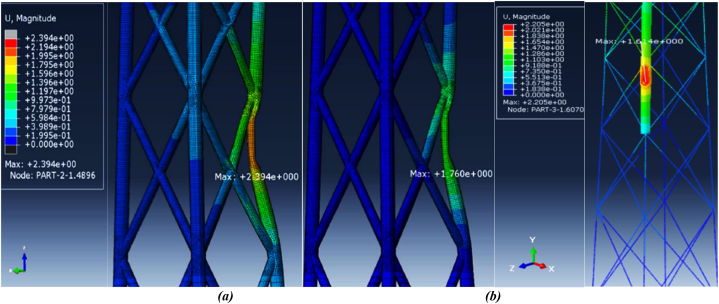
(a) Maximum displacement due to impact in Moulas et al.’s study [42]; **(b)** maximum displacement in the current simulation.

**Fig. 15:**
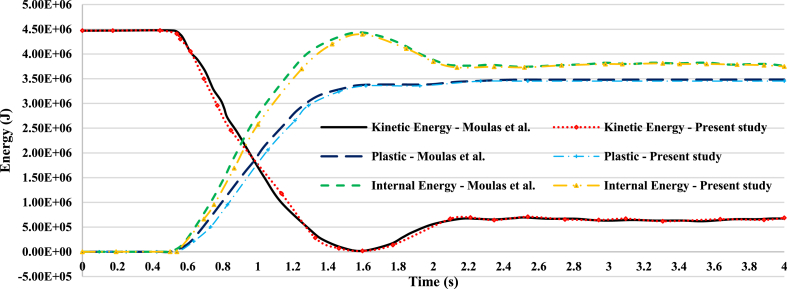
Compare plastic dissipation, kinetic energy, and internal energy with the study of Moulas et al.

In Moulas et al.'s modeling, the ship is a barge-type vessel with a displacement weight of 4000 tons, considered rigid. The validation conducted involves a side collision of the barge with the jacket structure at a 45-degree angle and a speed of 4 m/s. Following the numerical simulation of the ship collision with the offshore wind turbine structure and employing the approaches outlined in this study, the results were compared with those of Moulas et al. (2017), as detailed in Table 7. Additionally, a comparison of the maximum displacement is presented in Fig. 14(a-b). Due to the dynamic nature of the collision, the validation also considers the time history of energy transformation for a sideways collision between an offshore accommodation barge and a wind jacket foundation, with a collision angle of 0° and a ship velocity of 4 m/s (as shown in Fig. 15). Comparing the behavior of the energy conversion diagrams shows that the energy absorption in the case of the shell element jacket in Moulas et al.'s study is slightly higher than that in the combination of shell and beam elements in the present study. However, in general, the behavior of the structure is of acceptable accuracy.

**Table 7:** Comparison of the results of the current simulation for validation with the study of Moulas et al.

Validation Items	Moulas et al. Simulation [42]	Current Simulation
Maximum Displacement (*m*)	2.394	2.205
Residual Displacement (*m*)	1.760	1.614
Maximum S, Mises (*MPa*)	650	642.3

## 4 Numerical results and discussion

### 4.1 System energy conversation and stress analysis

**Fig. 16:**
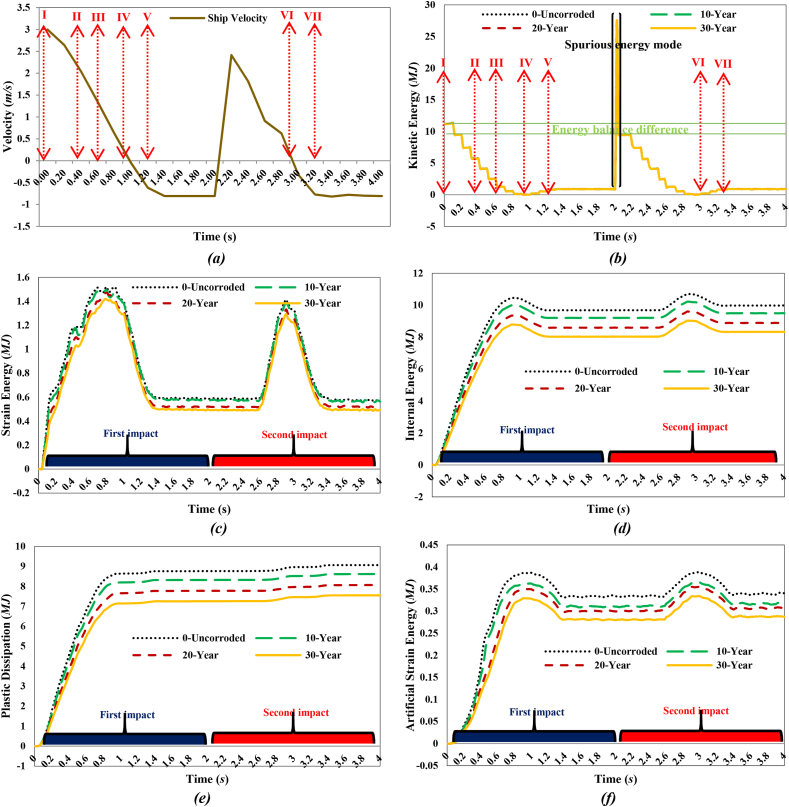
**(a)** Ship velocity and system energy conversation curves in forecastle collision with a leg: **(b)** kinetic energy; **(c)** strain energy; **(d)** internal energy; **(e)** plastic dissipation; **(f)** artificial strain energy.

In the event of a collision between a supply vessel and a jacket platform, the ship's kinetic energy is converted into various forms of energy, primarily internal energy, such as plastic deformation and strain energy in the jacket platform. During a collision, part of the kinetic energy is absorbed through plastic straining, structural motion and vibrations, and hydrodynamic dissipation by accelerating added masses. Depending on the collision angle and relative position, some kinetic energy may remain after the collision [45]. The conservation of energy between the jacket platform and the offshore supply vessel during a collision is crucial for assessing the accuracy of simulation results. Fig. 16(a) illustrates the ship's velocity, while Fig. 16(b–f) shows the curves depicting energy transformation during a collision involving a 2700-ton ship traveling at 3 m/s in a forecastle collision with a leg. The energy conversion curve can be classified into four distinct categories.

• **I**: When the supply vessel collides with the leg of the jacket platform, it creates an initial local indentation at the contact area, converting the vessel's kinetic energy into strain energy within the structure.
• **II-III**: Initially, the global response is elastic; however, at higher impact energies, the supply vessel continues to compress the jacket platform, exceeding allowable displacement limits and causing plastic deformation in certain members.
• **IV, VI**: The supply vessel stops displacing the jacket platform once it reaches a point of maximum indentation, with the vessel's kinetic energy being transformed into internal energy within the jacket.
• **V, VII**: A portion of the elastic energy stored in the jacket platform is released, repelling the striking supply vessel from the point of contact. The jacket platform then oscillates freely, utilizing residual internal energy until friction dissipates the remaining energy.

Based on Fig. 16(b-f), the initial kinetic energy of the supply vessel, approximately 11.2 MJ, gradually transforms into structural internal energy during the first impact. In the uncorroded case, this internal energy is 9.68 MJ, decreasing to 9.2 MJ after 10 years of service, 8.59 MJ after 20 years, and 8.02 MJ after 30 years, all measured at the end of 2 s. The kinetic energy decreases as the supply vessel contacts the jacket platform, reaching a minimum at 0.96 s in both the uncorroded and 10-year cases, and at 0.94 s in the 20- and 30-year scenarios. The return of kinetic energy after these times indicates that the ship reverses direction following the collision. As the ship prepares for the second collision and oscillates back and forth, there is a sudden peak in kinetic energy around 2 s due to spurious energy modes, resulting in a significant artificial oscillation in kinetic energy. This is further evidenced by a drop in artificial energy in the subsequent time step, leading to another collision with the platform. During this second impact, the initial kinetic energy is lower than in the first collision, as it is converted into internal energy within the system. Specifically, this internal energy amounts to 9.98 MJ in the uncorroded case, 9.5 MJ after 10 years of service, 8.89 MJ after 20 years, and 8.33 MJ after 30 years at the end of 4 s. As in the first impact, the kinetic energy decreases as the supply vessel recontacts the jacket platform during the second collision, reaching a minimum at 2.94 s in the uncorroded and 30-year cases, at 2.92 s in the 10-year scenario, and at 2.98 s in the 20-year case.

During both collisions, the trend of plastic dissipation aligns with the increase in internal energy, with the maximum ratio of plastic dissipation to internal energy gradually exceeding 90 %. This indicates that a significant portion of the kinetic energy is converted into plastic dissipation. To prevent hourglass modes that could cause severe distortions in the simulation, artificial energy was employed, with the goal of keeping it below 5 % of the internal energy [46]. Hourglass modes represent a non-physical type of deformation that results in zero strain and stress. In both collision scenarios, the maximum proportion of artificial energy to internal energy was 3.84 % in the uncorroded case, 3.76 % after 10 years, 3.81 % after 20 years, and 3.84 % after 30 years of service. These percentages indicate that the mesh density of the model and the calculation results are satisfactory from an energy perspective.

**Fig. 17:**
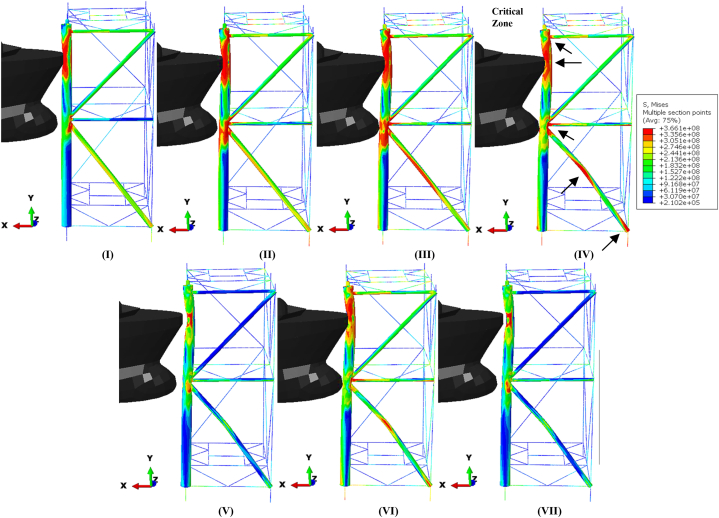
(I-VII). Time history of stresses of the jacket platform in the ship's forecastle collision with the leg in the uncorroded case.

Fig. 17(I-VII) illustrates the stress time history of the jacket platform during the ship's forecastle collision with the platform leg in the uncorroded case, categorized based on system energy conservation. The purpose of Fig. 17(I-VII) is to correlate the energy conversion diagrams with each stage of the collision between the ship and the jacket platform. As shown in Figs. 16(b) and Fig. 17(IV-V), at 0.96 s, the kinetic energy reaches its lowest level, indicating that the jacket leg experiences the most significant indentation. Subsequently, the return of kinetic energy after this point suggests that the ship reverses direction away from the damaged jacket, influenced by the initial strength of the ship's structure, as depicted in Fig. 17(IV-V). Additionally, Fig. 17(I-VII) examines the stress distribution in the jacket structure and identifies critical areas during each stage of the collision. In both collision events, the highest stress levels were observed in the impact zone, the connection region between the leg and bracing member, and near the middle of the bottom brace. At 0.96 s, when the kinetic energy reaches its lowest level during the first collision, the areas connecting the rear brace to the platform base and the middle section of the rear cross brace experience the highest stress. Notably, the maximum stress recorded was 366.1 MPa in these critical zones, surpassing the material's static yield stress. These vulnerable areas should be prioritized for protection and reinforcement during the platform design phase.

**Fig. 18:**
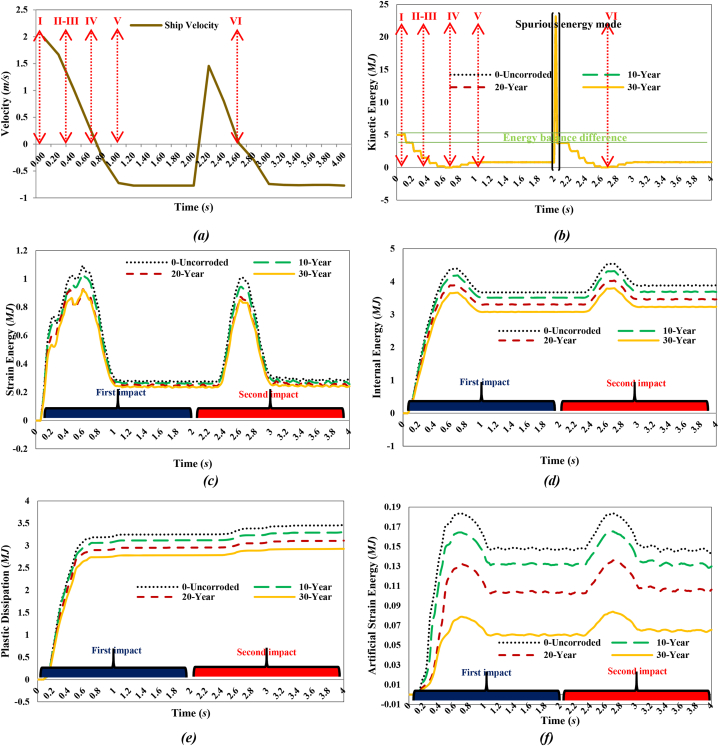
**(a)** Ship velocity and system energy conversation curves in sideway collision with a leg: **(b)** kinetic energy; **(c)** strain energy; **(d)** internal energy; **(e)** plastic dissipation; **(f)** artificial strain energy.

Fig. 18(a) shows the ship's velocity, while Fig. 18(b-f) illustrates the energy transformation curves of the jacket platform during a collision with a 2700-ton ship traveling at 2 m/s in a sideways impact with the platform's leg. The initial kinetic energy of the supply vessel, approximately 5 MJ, gradually transformed into structural internal energy. At the end of the first impact, the internal energy was measured at 3.67 MJ in the uncorroded case, 3.51 MJ after 10 years, 3.31 MJ after 20 years, and 3.08 MJ after 30 years of service. During the first collision, the kinetic energy decreased as the supply vessel made contact with the platform, reaching its minimum at 0.64 s in the uncorroded case, 0.7 s after 10 years, and 0.68 s after 20 and 30 years of service. The subsequent return of kinetic energy indicated that the ship moved in the opposite direction after the collision. As the vessel moved back and forth at the end of the first collision, its kinetic energy suddenly peaked due to spurious energy modes, resulting in significant artificial oscillations in kinetic energy. This is further evidenced by a drop in artificial energy in the subsequent time step, leading to another collision with the platform. Finally, it moved back toward the platform for a second collision. During the second collision, the initial kinetic energy was lower compared to the first impact. This kinetic energy was converted into the system's internal energy, resulting in 3.88 MJ in the uncorroded case, 3.69 MJ after 10 years, 3.46 MJ after 20 years, and 3.23 MJ after 30 years of service at the end of the second impact. The kinetic energy decreased during the second impact as the supply vessel made contact with the jacket again, reaching its minimum at 2.64 s in the uncorroded case, 2.62 s after 10 and 20 years, and 2.6 s after 30 years of service. During both collisions, the trend of plastic dissipation corresponded with the increasing trend of internal energy, with the maximum ratio of plastic dissipation to internal energy gradually rising to over 90 %, indicating that most of the kinetic energy was converted into plastic dissipation. Furthermore, during both collisions, the maximum proportion of artificial energy to internal energy was measured at 4.49 % in the uncorroded case, 4.2 % after 10 years, 3.6 % after 20 years, and 2.29 % after 30 years of service, indicating satisfactory mesh density and calculation results from an energy perspective.

**Fig. 19:**
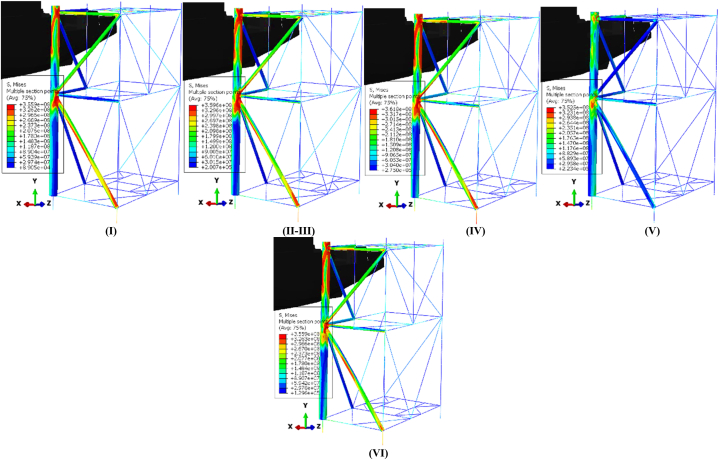
(I-VI). Time history of stresses of the jacket platform in the ship's sideway collision with the leg in the uncorroded case.

Fig. 19(I-VI) illustrates the temporal evolution of stresses on the jacket platform during a ship's sideways collision with one of the platform's legs, categorized based on energy transformation. It is important to note that the stress experienced by the impacted leg exceeded the yield stress threshold.

**Fig. 20:**
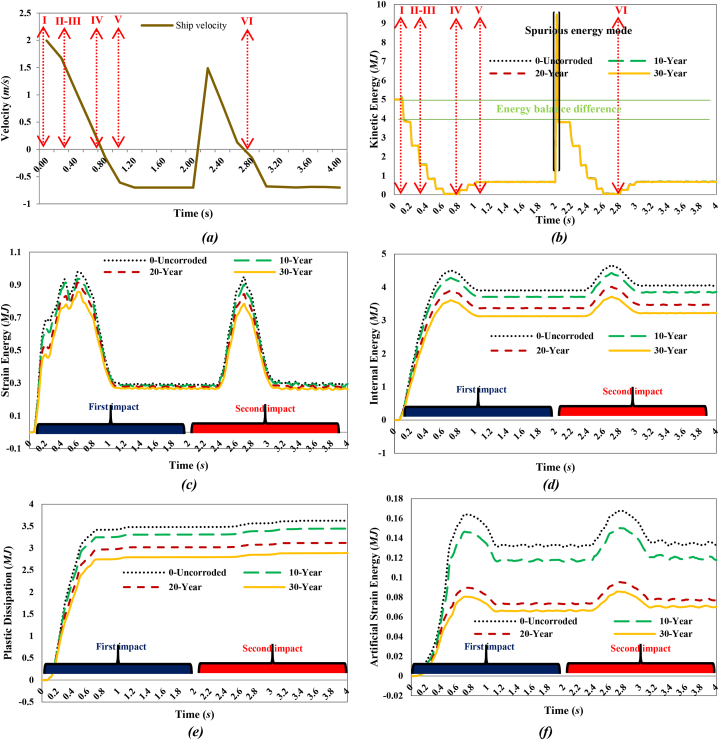
**(a)** Ship velocity and system energy conversation curves in stern collision with a leg: **(b)** kinetic energy; **(c)** strain energy; **(d)** internal energy; **(e)** plastic dissipation; **(f)** artificial strain energy.

Fig. 20(a) shows the ship's velocity, while Fig. 20(b-f) illustrates the energy transformation curves of the jacket platform during a collision with a 2700-ton ship traveling at 2 m/s in a stern collision with the platform's leg. Initially, the kinetic energy of the supply vessel is approximately 5 MJ and gradually transforms into structural internal energy during the collision. In the uncorroded case, the internal energy reaches 3.91 MJ, decreasing to 3.71 MJ after 10 years of service, 3.37 MJ after 20 years, and 3.13 MJ after 30 years. During the first impact, the kinetic energy decreases as the vessel contacts the platform, reaching its minimum at 0.74 s in all scenarios. The subsequent increase in kinetic energy indicates that the ship moves away from the platform before returning for a second collision with similar energy levels. In this second collision, the kinetic energy is converted into internal energy, with values of 4.06 MJ in the uncorroded case, 3.86 MJ after 10 years, 3.48 MJ after 20 years, and 3.22 MJ after 30 years. Plastic dissipation corresponds with the growth of internal energy during both collisions, with the maximum ratio of plastic dissipation to internal energy exceeding 89 %, indicating that most of the kinetic energy is converted into plastic dissipation. The proportion of artificial energy to internal energy peaks at 3.83 % in the uncorroded case, 3.57 % after 10 years, 2.48 % after 20 years, and 2.41 % after 30 years, suggesting satisfactory mesh density and calculation accuracy in terms of energy distribution.

**Fig. 21:**
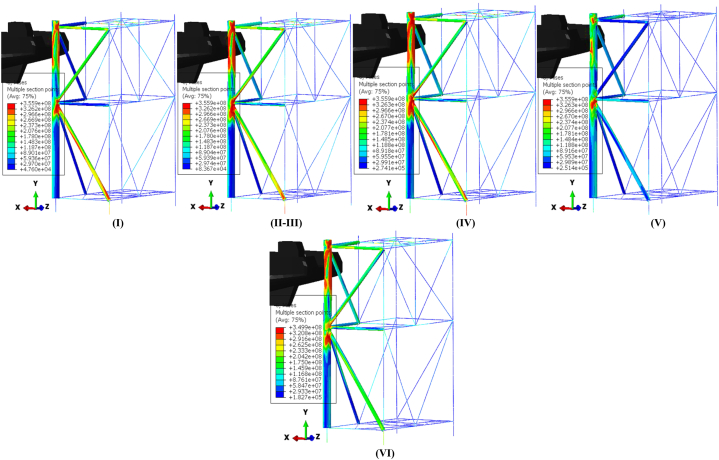
(I-VI). Time history of stresses of the jacket platform in the ship's stern collision with the leg in the uncorroded case.

Fig. 21(I-VI) displays the time history of stresses on the jacket platform resulting from the collision of the ship's stern with the leg of the jacket, categorized based on the four types of energy transformation. It is worth noting that the stresses at the impacted leg and within the collision zone exceeded the yield stress.

**Fig. 22:**
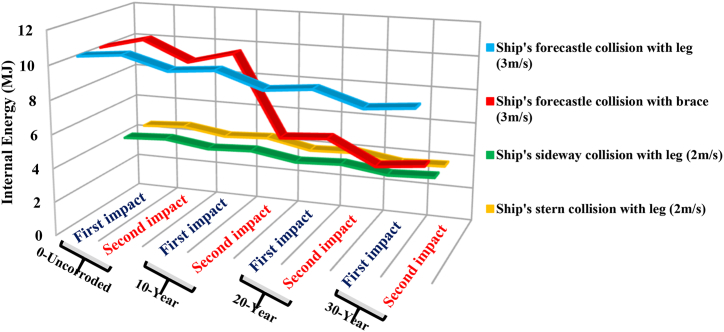
Trend line of maximum energy in the whole model for each collision scenario.

The trend line of maximum internal energy patterns for all collision scenarios, presented in Fig. 22, indicates that at the same impact speed of 3 m/s, and up to a corrosion rate of 10 years, the energy of the jacket in the cases of the ship's forecastle collision with the leg and bracing member does not change significantly. However, after the 10-year corrosion rate, the energy absorption of the jacket in collisions with the bracing member becomes much lower compared to collisions with the leg of the platform. This suggests that when the impacted brace fails, it can no longer absorb energy, leading to reduced energy dissipation in the jacket. At the same impact speed of 2 m/s, the internal energy of the jacket in the sideways impact and stern collision scenarios of the ship are not significantly different from each other. However, the internal energy level is slightly higher in the stern collision scenario because more legs of the jacket platform are deformed due to the impact. The figure indicates that with an increase in displacement of the jacket platform, the internal energy also increases.

### 4.2 Strength analysis of jacket platform

**Fig. 23:**
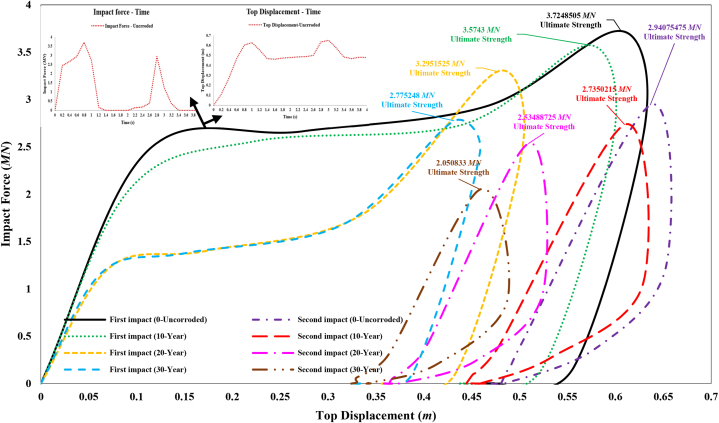
Comparison of ultimate strength of jacket platform during the ship's forecastle collision with leg.

In this study, the ultimate strength of the jacket platform is assessed using force-displacement curves. Fig. 23 illustrates the impact force versus top displacement curve during a ship's forecastle collision with the leg, both in the absence of defects and with corrosion defects. Initially, upon collision, the top displacement of the jacket rapidly increases with a significant force. Subsequently, the displacement continues to increase with less force until reaching the structure's ultimate strength. As the collision force reaches its peak, the ship gradually moves away from the jacket platform, and the force diminishes until complete separation occurs, stabilizing the displacement at a permanent value. The ultimate strength of the jacket without corrosion defects, upon initial impact, is approximately 3.72 MN. With corrosion defects corresponding to 10-year, 20-year, and 30-year periods, the strength decreases by 4 %, 12 %, and 25 %, respectively.

According to Fig. 23, the collision force experienced by the jacket in the second collision is reduced compared to the first collision due to the decreased strength of the structure. When the platform reaches its maximum ultimate strength in the second collision, the hardness of the jacket and the energy dissipated through local denting and bending of the leg cause the force level to diminish until it stabilizes at its permanent displacement value, accompanied by overall structural vibration. The ultimate strength of the jacket without corrosion defects, after the secondary impact, is approximately 2.94 MN, representing a 21.05 % decrease compared to the state without defects and after the primary impact. With corrosion defects corresponding to 10-year, 20-year, and 30-year periods, the ultimate strength decreases by 26.57 %, 31.95 %, and 44.94 %, respectively, compared to the intact state. Additionally, as shown in Fig. 23, with increasing corrosion thickness, the maximum jacket strength is reached at a lower top displacement. However, the secondary impact results in an increased top displacement compared to the primary impact, even though the ultimate strength of the platform decreases.

**Fig. 24:**
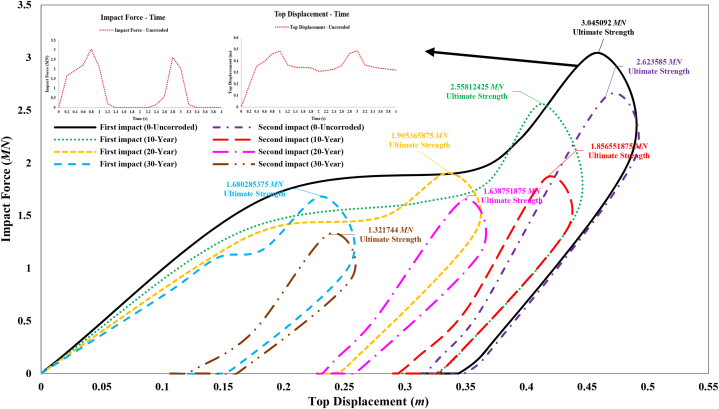
Comparison of ultimate strength of jacket platform during the ship's sideway collision.

**Fig. 25:**
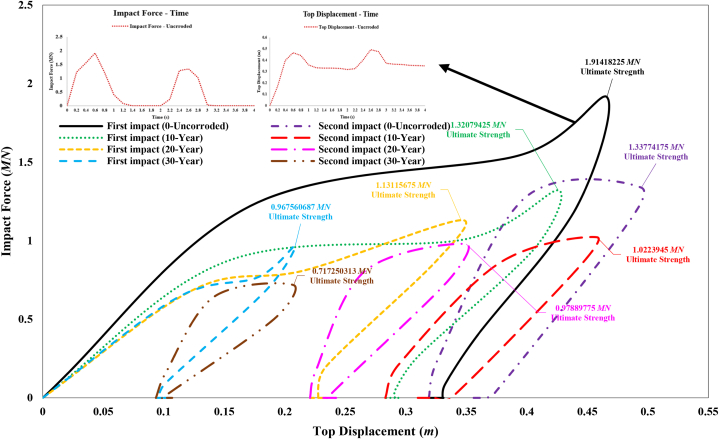
Comparison of ultimate strength of jacket platform during the ship's stern collision.

Fig. 24 shows the impact force versus jacket top displacement curve during a ship's sideways collision with the leg of the fixed jacket platform, both without defects and with corrosion defects. The ultimate strength of the jacket without corrosion defects, after the initial impact, is approximately 3.05 MN. This strength is reduced by 15.99 %, 37.43 %, and 44.82 % when considering corrosion states of 10-year, 20-year, and 30-year periods, respectively. After the secondary impact, the ultimate strength of the jacket without corrosion defects is about 2.62 MN, which represents a 13.84 % decrease compared to the state after the primary impact. With corrosion states of 10-year, 20-year, and 30-year periods, the ultimate strength further decreases by 39.03 %, 46.18 %, and 56.59 %, respectively, compared to the intact state. As expected, the structural strength decreases with increasing corrosion rates and the application of secondary impacts. Fig. 25 shows the impact force versus jacket top displacement curve during a ship's stern collision with the leg of the fixed jacket platform, both without defects and with corrosion defects. The ultimate strength of the jacket without corrosion defects, after the initial impact, is approximately 1.91 MN. This strength is reduced by 31 %, 40.9 %, and 49.45 % for corrosion states of 10-year, 20-year, and 30-year periods, respectively. After the secondary impact, the ultimate strength of the jacket without corrosion defects is about 1.34 MN, representing a 30.11 % decrease compared to the state after the primary impact. With corrosion states of 10-year, 20-year, and 30-year periods, the ultimate strength further decreases by 46.59 %, 48.86 %, and 62.52 %, respectively, compared to the intact state. As expected, the structural strength decreases with increasing corrosion rates and the application of secondary impacts.

**Fig. 26:**
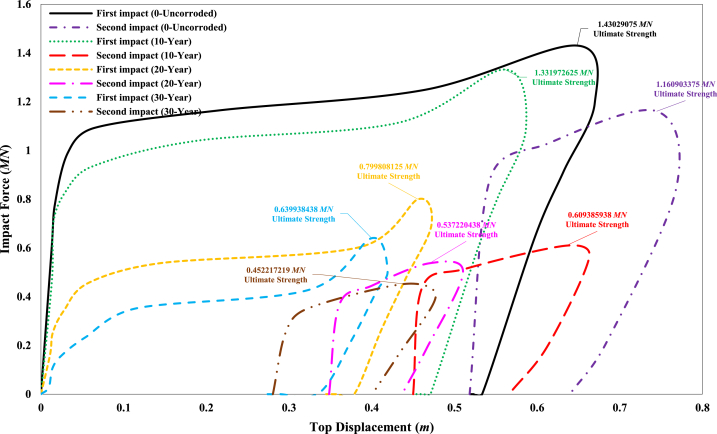
Comparison of ultimate strength of jacket platform during the ship's forecastle collision with the brace member.

Fig. 26 presents the impact force versus top displacement curve of the jacket for the collision of the ship's forecastle with the bracing member of the fixed jacket platform under both intact and corroded conditions. The ultimate strength of the jacket without corrosion defects under the initial impact is approximately 1.43 MN. This strength is reduced by 6.87 %, 44.08 %, and 55.26 % for corrosion scenarios representing 10-year, 20-year, and 30-year periods, respectively. For the secondary impact, the ultimate strength of the jacket in the non-corroded state is about 1.16 MN, an 18.83 % decrease compared to the intact state under the primary impact. The secondary impact further reduces the ultimate strength by 57.4 %, 62.43 %, and 68.38 % for the 10-year, 20-year, and 30-year corrosion scenarios, respectively, compared to the intact state. As expected, the jacket's strength decreases with increasing corrosion rates and the application of the secondary impact. The results clearly indicate that when the ship structure collides with a bracing member, the jacket platform exhibits significantly lower strength than when it collides with a leg. Additionally, the results show a greater reduction in platform strength during the second impact for each corrosion scenario compared to the first impact in the 10-year corrosion scenario. This suggests that, in collisions between the ship's forecastle and the jacket, the secondary impact has a more pronounced effect on platform strength than the reduction in member thickness due to increased corrosion.

Based on the global structural response, the area under the force-displacement curve represents the energy absorption of the offshore jacket structure during a ship collision. A general examination of the diagrams for all collision scenarios with different impact locations indicates that corrosion and secondary impacts significantly reduce the energy absorption during the collision (as indicated by the reduction in the area under the curve) and the global strength of the structure. At the same impact speed of 3 m/s, in the scenario of a forecastle collision with the leg, the jacket structure demonstrates better impact energy absorption compared to the forecastle collision with the bracing member. This suggests that the local stiffness of the damaged member plays a crucial role in the overall energy absorption. In the forecastle collision with the bracing member, the primary behavior of the structure occurs in the plastic region, quickly transitioning from the elastic region with minimal displacement and immediately entering the plastic region. The structural capacity curves in the side and stern collisions of the ship with the platform leg exhibit similar behavior, with the platform displacing significantly—about 0.15 m—beyond the linear region and entering the plastic region.

### 4.3 Local deformation analysis

**Fig. 27:**
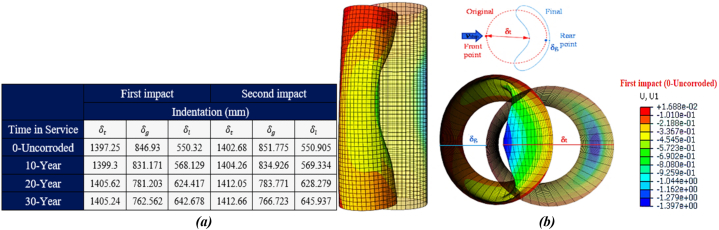
(a) Comparison of local deformation of the leg during the ship's forecastle collision; **(b)** leg deformation.

Two impact indices were proposed in this study to estimate the deformation of the jacket platform: local displacement ($\delta_{t}$) and global bending deformation value ($\delta_{g}$). According to Zhang et al. [47], if ($\delta_{t}$) represents the total displacement at the point of collision, local indentation ($\delta_{l}$) can be calculated as ($\delta_{l}$ = $\delta_{t}$ - $\delta_{g}$). Fig. 27(a-b) illustrates the indentation of the leg of the jacket platform during a ship's forecastle collision. Initially, indentation forms at the onset of the collision. As the collision force increases, the adjacent bracing members exhibit stiffness in the connection zone to the leg, preventing it from bending inward. This results in increased indentation in the collision area and squashing in the zone where the bracing member connects to the leg. Additionally, the tubular member loses its circular shape and deforms into an oval shape. The leg of the platform also undergoes buckling and bending, although the force applied to the tubular member is not significant enough to cause contact between the inner walls of the leg.

**Fig. 28:**
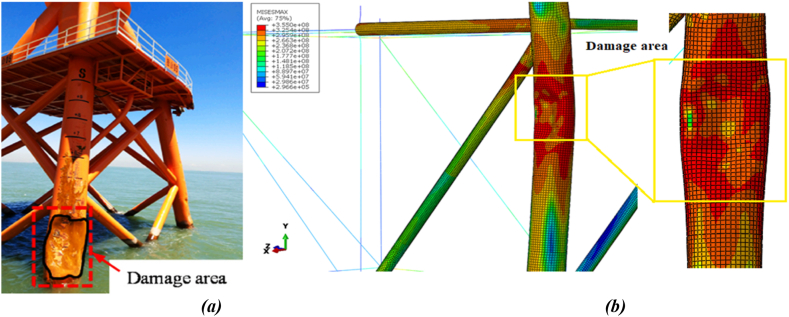
(a) Actual damage [48]; **(b)** Simulated damage to the jacket during the ship's forecastle collision with the leg.

As shown in the table in Fig. 27(a), the local indentation of the leg is 550.32 mm in both the uncorroded state and the initial impact state of the ship's forecastle against the platform. With a 30-year corrosion rate, this indentation increases to about 643 mm. After applying a secondary impact, the indentation is 550.905 mm in the uncorroded state and reaches about 646 mm with a 30-year corrosion rate. In all scenarios, the global bending deformation exceeds the local indentation. Although corrosion has reduced the overall thickness of structural members, it has led to decreased overall bending deformation and increased total displacement, resulting in greater local indentation. Fig. 28(a-b) also shows the comparison of damage to the leg in the actual and simulated states.

**Fig. 29:**
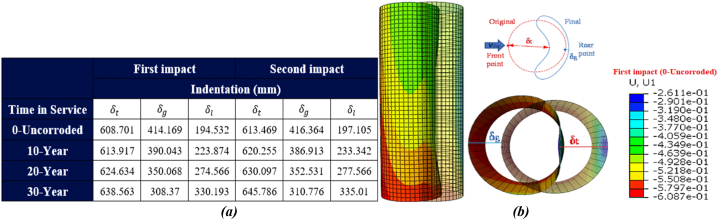
(a) Comparison of local deformation of the leg during the sideway collision; **(b)** Leg deformation.

Fig. 29(a-b) shows the gradual indentation of the leg during the ship's sideways collision. The tubular member experiences three modes of failure: buckling, bending, and deformation into an oval shape. As illustrated in Fig. 29 (a), the local indentation of the leg measures 194.532 mm in both the uncorroded state and the initial impact state of the ship's sideways collision against the platform. With a 30-year corrosion rate, this indentation increases to approximately 330.93 mm. After a secondary impact, the indentation is 197.105 mm in the uncorroded state and reaches about 335.01 mm with a 30-year corrosion rate. Unlike forecastle collisions, the reduction in collision velocity and the smoother shape of the ship's hull result in less local deformation.

**Fig. 30:**
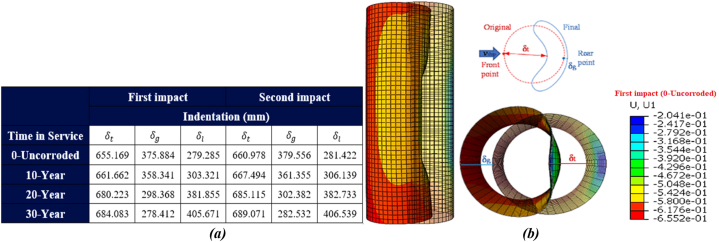
**(a)** Comparison of local deformation of the leg during the ship's stern collision; **(b)** Leg deformation.

Fig. 30(a-b) shows the local displacement of the leg during the ship's stern collision. Unlike the sideways collision, where the same collision velocity is considered, the impact force is lower, but the amount of indentation is greater. This indicates that at the same impact velocity, the shape of the impacting object significantly affects the extent of indentation. The local indentation of the leg measures 279.285 mm in both the uncorroded state and the initial impact state of the ship's stern collision against the platform. With a 30-year corrosion rate, this indentation increases to approximately 405.671 mm. After a secondary impact, the indentation is 281.422 mm in the uncorroded state and reaches about 406.539 mm with the 30-year corrosion rate.

**Fig. 31:**
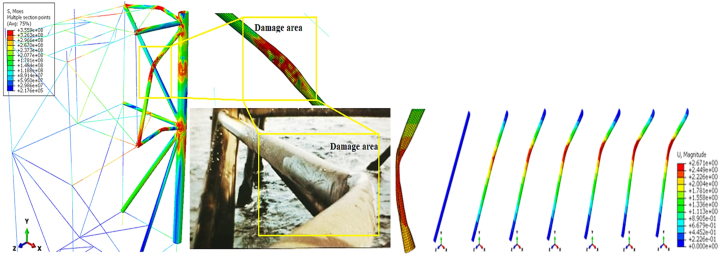
Local deformation of the bracing member during the ship's forecastle collision.

Fig. 31 shows the local deformation of the bracing member during the ship's forecastle collision. As the impact force increases, in addition to local indentation and squashing at the connection areas, the bracing member between the two legs of the jacket bends to limit excessive displacement. Furthermore, the impacted brace undergoes buckling and bending modes. Unlike the forecastle collision with the leg, in this collision scenario, the tubular member also experiences contact mode, where the inner walls of the bracing member come into contact with each other. Fig. 31 also compares the damage to the bracing member in the actual and simulated states.

### 4.4 Structural damage analysis

**Fig. 32:**
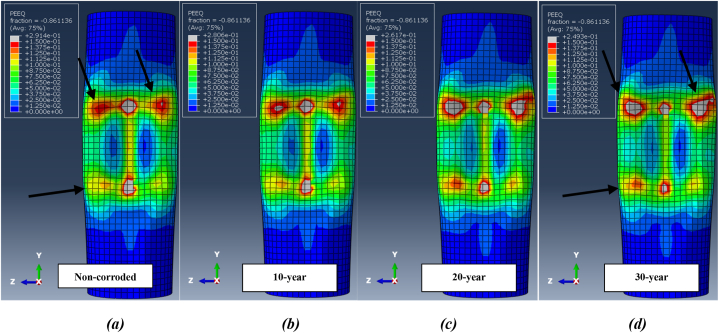
Distribution of plastic strain at the collision zone after two successive ship's forecastle impacts for each corrosion scenario: **(a)** Non-corroded; **(b)** 10-Year; **(c)** 20-Year; **(d)** 30-Year.

The damaged deformation of the jacket platform is indicated by plastic strain. Fig. 32(a-d) illustrates the distribution of plastic strain on the leg at the collision zone after two successive impacts for each corrosion scenario during a ship's forecastle collision. The figure shows that the impact zone is particularly vulnerable and has experienced plastic deformation, influenced by the shape of the ship's forecastle. Comparing plastic failure areas reveals that as the wall thickness of tubular members decreases due to corrosion, the extent of plastic strain in the impact zone increases, causing the elements to reach plastic strain more quickly. However, the overall amount of plastic strain decreases with higher corrosion rates. The values of plastic strain at the impact zone of the leg after two successive impacts during a ship's forecastle collision are 0.291 for the uncorroded state, 0.280 after 10 years of service, 0.261 after 20 years, and 0.249 after 30 years. Additionally, the application of a secondary impact exacerbates collision damage, increasing the plastic strain. The plastic failure area continues to expand until the ship separates from the jacket platform.

**Fig. 33:**
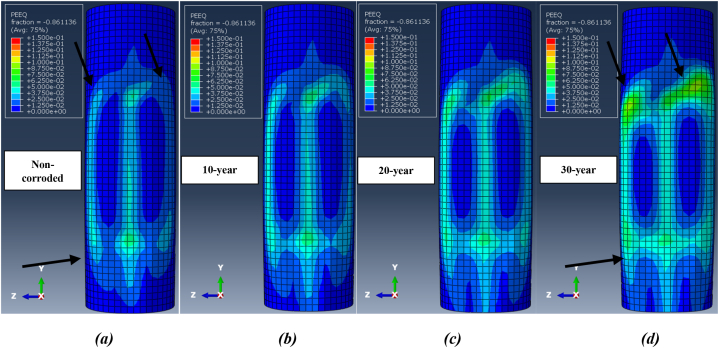
Distribution of plastic strain at the collision zone after two successive ship's sideway impacts for each corrosion scenario: **(a)** Non-corroded; **(b)** 10-Year; **(c)** 20-Year; **(d)** 30-Year.

**Fig. 34:**
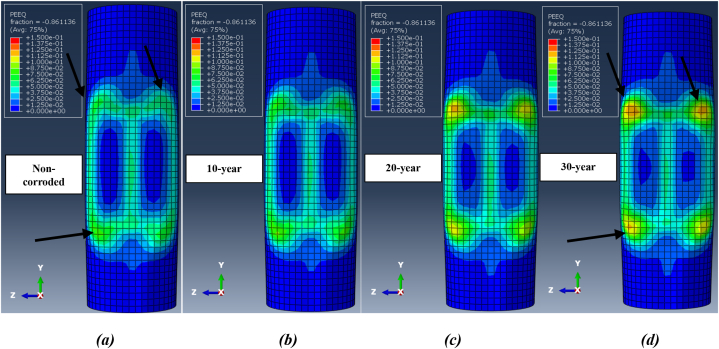
Distribution of plastic strain at the collision zone after two successive ship's stern impacts for each corrosion scenario: **(a)** Non-corroded; **(b)** 10-Year; **(c)** 20-Year; **(d)** 30-Year.

Fig. 33(a-d) shows the distribution of plastic strain at collision zones after two impacts during a ship's sideways collision with the leg. Similar to the ship's forecastle collision scenario, an increased number of collisions and a higher corrosion rate result in a larger area of collision damage. Reducing the wall thickness of all members in the jacket, based on the corrosion model, effectively decreases the numerical value of plastic strain, as the elements undergo deformation faster and show less resistance. Due to the lower collision velocity and the smoother shape of the ship's hull in this scenario, the amount of plastic deformation is less than in the ship's forecastle impact scenario. Fig. 34(a-d) shows the distribution of plastic strain in the critical zone after successive impacts during a ship's stern collision. At the same initial collision velocity of 2 m/s, the amount of damage in this scenario is greater than that in the ship's sideways collision, which is attributed to the difference in the shape of the impacting structure.

## 5 Conclusions and potential topics for future research

In this paper, the strength of an offshore jacket platform located in the Persian Gulf was evaluated using the finite element method in ABAQUS software. The evaluation focused on the platform's response to two consecutive collisions with a 2700-ton offshore supply vessel. A total of 16 collision scenarios were simulated and analyzed, varying by corrosion rate (based on Yang et al.'s study) and the collision zone on the ship's structure. Several conclusions were drawn from the collision simulation results.

**Fig. 35:**
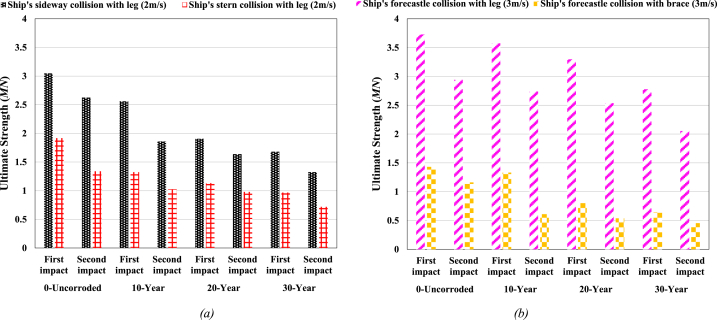
Comparison of ultimate strength in all scenarios; **(a)** ship's sideway and stern collision; **(b)** Ship's forecastle collision.

The modeling results highlight the effects of secondary impacts and corrosion rates as follows: In the side and stern collisions, the primary collision in each corrosion scenario has nearly the same effect on the ultimate strength of the structure as the secondary collision in the preceding corrosion scenario (Fig. 35(a)). However, in forecastle collisions with both the leg and bracing member, the secondary impact has a greater effect than the primary impact in the subsequent corrosion scenario, equivalent to a 10-year increase in corrosion (according to Fig. 35(b)). For example, in the scenario where the ship's forecastle collides with the leg, there is a 48.86 % reduction in strength in the second impact with the 20-year corrosion scenario, compared to a 49.45 % reduction in the first impact with the 30-year corrosion scenario in the stern collision with the leg. Additionally, the results reveal that, at the same impact velocity, the jacket platform exhibits significantly less strength when the ship's stern collides with the leg compared to a sideways collision. Furthermore, at the same collision velocity of 3 m/s, when the ship structure collides with the bracing member, the jacket platform has much less strength than when it collides with the leg.

The key factors affecting structural damage are corrosion rate, consecutive impacts, and the shape of the impacting structure. As the wall thickness of the tubular members decreases due to corrosion, the extent of plastic strain in the impact zone increases. The application of a secondary impact exacerbates the collision damage, increasing plastic strain and expanding the plastic failure area until the ship separates from the platform. In the case of the ship's forecastle impact on the leg, due to the higher impact speed and sharper shape, the plastic strain is the highest. At the same speed, in the stern and side impacts, the amount of plastic deformation is lower in the side impact due to the smoother body shape.

Based on the three impact indices proposed to estimate the deformation of the jacket's leg, the findings reveal that in all scenarios the local indentation ($\delta_{l})$ and local deformation ($\delta_{t})$ indices are directly related to increases in the corrosion rate and the number of collisions. However, the bending deformation parameter ($\delta_{g}$) exhibits a slightly different pattern: it has an inverse relationship with the corrosion rate and a direct relationship with the number of collisions. At the same collision speed and under the same corrosion scenarios, the local indentation index is higher in the ship's stern collision mode, whereas the bending deformation index has larger values in the side collision mode. Forecastle collisions result in significant local displacement. Finally, system energy conservation reveals that energy absorption has an inverse relationship with increasing corrosion rates and a direct relationship with the increasing number of collisions.

The scope of this research is limited. Future work will address soil-structure and wave-structure interactions on the jacket platform during ship collision analysis. It is also recommended to model the ship as a deformable structure to evaluate the strength of the ship's structure. Additionally, future studies will consider the effects of pitting corrosion to assess the strength of the jacket platform. Developing new analytical relations that incorporate all relevant parameters, including impact zone characteristics, boundary conditions, and other critical factors, is also recommended for future work.

**Table:** List of symbols

*d(t)*	: Corrosive depth	τ	: Time interval after the appearance of progressive pitting points (years)
***T***	: Time-in-service (years)	***a*** and ***b***	: Parameters that control the corrosion process
***z(t)***	: Pitting depth	$T_{c}$	: Life of coating (years)
$d_{\infty }$	: Long-term corrosion wastage	$T_{t}$	: Transition time (years)
$C_{1}$	: Corrosion rate coefficient	$C_{2}$	: Corrosion pattern coefficient
$t_{0}$	: Initial thickness of the tube	$T_{cl}$	: General corrosion initiation time (years)
${Cr}_{\max}$	: Maximum corrosion depth	$t\left(\theta_{1}\right)$	: Thickness of the section of the member at angle $\theta_{1}$
***LAT***	: Lowest astronomical tide	***HAT***	: Highest astronomical tide
$\delta_{g}$	: Global bending deformation	$\delta_{t}$	: Local displacement
$\delta_{l}$	: Local indentation		

## CRediT authorship contribution statement

**Reza Zendehdel:** Writing – original draft, Software, Investigation, Conceptualization. **Mohammad Reza Khedmati:** Writing – review & editing, Supervision, Resources.

## Data and code availability statement

The authors do not have permission to share data.

## Funding information

This research did not receive any specific grant from funding agencies in the public, commercial, or not-for-profit sectors.

## Declaration of competing interest

The authors declare that they have no known competing financial interests or personal relationships that could have appeared to influence the work reported in this paper.
